# Larval and adult environmental temperatures influence the adult reproductive traits of *Anopheles gambiae* s.s.

**DOI:** 10.1186/s13071-015-1053-5

**Published:** 2015-09-17

**Authors:** Céline D. Christiansen-Jucht, Paul E. Parham, Adam Saddler, Jacob C. Koella, María-Gloria Basáñez

**Affiliations:** Department of Infectious Disease Epidemiology, School of Public Health, Faculty of Medicine (St Mary’s campus), Imperial College London, Norfolk Place, London, W2 1PG UK; Department of Public Health and Policy, Faculty of Health and Life Sciences, University of Liverpool, 33 Finsbury Square, London, EC2A 1AG UK; Department of Epidemiology and Public Health, Health Interventions Unit, Swiss TPH, Basel, Switzerland; Faculté des Sciences, Institut de Biologie, Université de Neuchâtel, Rue Emile-Argand 11, CH-2000 Neuchâtel, Switzerland

**Keywords:** *Anopheles gambiae sensu stricto*, Environmental temperature, Larval size, Wing length, Development, Fecundity, Fertility, Blood feeding, Climate change

## Abstract

**Background:**

*Anopheles* mosquito life-history parameters and population dynamics strongly influence malaria transmission, and environmental factors, particularly temperature, strongly affect these parameters. There are currently some studies on how temperature affects *Anopheles gambiae* s.s. survival but very few exist examining other life-history traits. We investigate here the effect of temperature on population dynamics parameters.

**Methods:**

*Anopheles gambiae* s.s. immatures were reared individually at 23 ± 1 °C, 27 ± 1 °C, 31 ± 1 °C, and 35 ± 1 °C, and adults were held at their larval temperature or at one of the other temperatures. Larvae were checked every 24 h for development to the next stage and measured for size; wing length was measured as a proxy for adult size. Females were blood fed three times, and the number of females feeding and laying eggs was counted. The numbers of eggs and percentage of eggs hatched were recorded.

**Results:**

Increasing temperatures during the larval stages resulted in significantly smaller larvae (*p* = 0.005) and smaller adults (*p* < 0.001). Adult temperature had no effect on the time to egg laying, and the larval temperature of adults only affected the incubation period of the first egg batch. Temperature influenced the time to hatching of eggs, as well as the time to development at every stage. The number of eggs laid was highest when adults were kept at 27 °C, and lowest at 31 °C, and higher adult temperatures decreased the proportion of eggs hatching after the second and third blood meal. Higher adult temperatures significantly decreased the probability of blood feeding, but the larval temperature of adults had no influence on the probability of taking a blood meal. Differences were observed between the first, second, and third blood meal in the times to egg laying and hatching, number of eggs laid, and probabilities of feeding and laying eggs.

**Conclusions:**

Our study shows that environmental temperature during the larval stages as well as during the adult stages affects *Anopheles* life-history parameters. Data on how temperature and other climatic factors affect vector life-history parameters are necessary to parameterise more reliably models predicting how global warming may influence malaria transmission.

**Electronic supplementary material:**

The online version of this article (doi:10.1186/s13071-015-1053-5) contains supplementary material, which is available to authorized users.

## Background

Climate change is expected to have a potentially important influence on the spread and transmission of vector-borne diseases, including mosquito-borne diseases such as malaria While shifts in environmental factors associated with climate change are expected to influence the global and/or local spread of malaria [1, 2], our knowledge of the precise effect of climatic factors, including temperature, on disease transmission remains limited [3, 4]. There are two main reasons for our lack of understanding.

Firstly, environmental temperature will play a complex role in the transmission of vector-borne diseases by influencing many life-history traits of both the mosquitoes and parasites that underlie transmission. It is clear, for example, that increasing mean temperatures associated with climate change may shorten the life span of the malaria vector [5, 6], and increase the developmental rate of the parasite within the mosquito [7–9], affecting transmission in opposing ways [10, 11]. It is also clear that higher temperature during larval development increases developmental rate and decreases the size of adult mosquitoes (and other cold-blooded animals) [12, 13]. Size of the mosquito can affect other parameters important for transmission such as fecundity [14, 15] (which affects mosquito population density), mating probability [16], or blood meal size [14, 17], which will be correlated with the uptake of parasites, and therefore with the probability of infection and transmission, as well as longevity and survival [18]. Mean environmental temperatures and temporal temperature fluctuations, as well as other climatic factors such as rainfall and humidity, have been shown to impact the dynamics of *Anopheles* vector populations [19, 20]. A better understanding of the effects of environmental factors on mosquito ecology, and the quantification of these effects, are therefore extremely valuable for predictions of vector abundance, distribution, evolutionary fitness, and transmission capacity [11, 21], and thereby the spread of malaria, particularly under the influence of climate change.

Secondly, the effects of temperature are usually studied during either larval development [22–24] (influencing the rate of development) or adult life (influencing life span [25]), but while it has been shown that temperatures during these two life stages can interact to influence important life-history traits such as longevity [26], no data currently exist, to the best of our knowledge, on the effect of environmental temperature to which immature stages of *Anopheles gambiae* Giles sensu stricto (s.s.) are exposed on the life and reproductive traits of the adult mosquito. A clearer and more comprehensive understanding of these interactions will allow more accurate predictions of how temperature can affect the ecology and population dynamics of the malaria vector, and thus, in turn, malaria transmission.

We present here results on the effects of environmental temperature during the larval and adult stages of *Anopheles gambiae* s.s. on several of its life-history traits; results on how temperature impacts survival have been presented elsewhere [26].

## Methods

### Larval maintenance and temperature regimes

*Anopheles gambiae* s.s. from the Kisumu colony from Western Kenya, were maintained at Imperial College London’s Silwood Park campus, by blood feeding on JCK and AS’s arms. After hatching, each larva was held in 3 ml of deionised water in one well of a 12-well plate at either 23 ± 1 °C, 27 ± 1 °C, 31 ± 1 °C, or 35 ± 1 °C (air temperature). An equal number of larvae (*n* = 640) were placed at each temperature and fed TetraMin® baby fish food according to the regimen described in [26]. As the sex of the larvae cannot be identified, we assumed a ratio of 1:1 of males to females. Larvae reared at 35 °C all died before emergence into adults. Both larvae and adults were reared in a 12:12 light/dark cycle and at 75 % (±5 %) relative humidity (RH).

#### Larval developmental rates and larval size

Larvae were examined for emergence to the next developmental stage (from L1 to L2, L2 to L3, L3 to L4, L4 to pupae, and pupae to adults), and photographed every 24 h to record developmental rates and larval size for seven days until all larvae pupated. The photographs were analysed and the larvae measured with the software ImageJ (ImageJ64 1.45 s, NIH, USA). Larval body length was measured from the distal tip of the head to the end of the anal segment excluding all antennae, feeding brush, and caudal hair. Larval head width (for instar determination) was measured across the widest part of the head (Additional file 1: Figure S1).

### Adult maintenance and temperature regimes

In order to distinguish between the effects of larval and adult environmental temperatures, and their possible interaction, the adult mosquitoes were divided into three batches immediately upon emergence, and each batch was held in a cage at either 23 ± 1 °C, 27 ± 1 °C, or 31 ± 1 °C (as described in [26]). Adults were allowed to mate for four days, following which all males were discarded. The females were then held individually in plastic cups and fed with cotton balls soaked with a 10 % sugar solution, which were discarded 24 h before each blood meal. The females were offered blood meals on CC-J’s arm at three time points: 5, 12, and 19 days after emergence in order to allow for egg laying.

#### Feeding, oviposition and egg hatching

The number of females feeding at each blood meal was counted, and mosquitoes that did not feed were thereafter not included in the experiment. The proportion of females imbibing blood at each blood meal and each of the adult environmental temperatures was recorded.

The bottom of each cup was filled with deionised water 24 h after each blood meal to encourage oviposition, and the adult mosquitoes were transferred to new, dry cups 48 h after laying eggs. Eggs were kept at the same environmental temperature as their mothers until hatching. The number of females that laid eggs after blood feeding, the proportion of egg-laying females among those which blood fed, the number of eggs laid by such females (a measure of fecundity), and the number of eggs that hatched among those laid (a measure of fertility) were counted. The time between blood feeding and egg laying and the time between egg laying and hatching were also recorded. Censoring occurred 35 days after the emergence of the parental generation into L1 larvae, with all mosquitoes monitored until that day (as described in [26]).

#### Adult female size

On day 35 after the parental generation emerged as L1 larvae, mosquitoes still alive were frozen and all adult females were measured. Both wings of each mosquito were removed, glued onto a microscope slide, photographed, and measured from the distal end of the alula to the tip with the software ImageJ (Additional file 1: Figure S2). As mosquitoes were not followed until their natural death, longer-lasting effects of the temperature of the maternal environment may have been lost. Data on the effect of temperature during the immature and mature stages on mosquito survival have been presented elsewhere [26] and will not be considered further here.

### Statistical methods

#### Larval developmental rates and larval size

A non-parametric (Kaplan-Meier) survival analysis was performed on the time for each mosquito stage to develop to the next larval, pupal, or adult stage, to allow for a comparison of how different temperature regimens during the immature stages affected the time to development from L1 to L2, L2 to L3, L3 to L4, L4 to pupae (P), and pupae to adults (A). The number of cumulative days to development for the L1–L2, L2–L3, L3–L4, L4–P, and P–A transitions was also calculated. Larval body size was measured from the distal tip of the head, excluding antennae, feeding brush, and caudal hair, to the end of the anal segment [27]. For each larval instar, the arithmetic mean of larval sizes was taken. Tukey’s test was used to compare mean instar-specific larval sizes between pairs of larval temperature regimens. For each of the seven days the larvae were followed to pupation, the arithmetic mean of larval sizes across all larval stages (from L1 to L4) were compared across all four larval temperatures using the F statistic.

#### Blood feeding and oviposition

The effect of larval and adult environmental temperature on the probability of female mosquitoes taking blood meals (measured as the proportion of females imbibing blood from the total that were offered a blood meal at each adult temperature), and on the probability of oviposition after each blood meal (measured as the proportion of females ovipositiong among those which had), was analysed by logistic regression and compared using the log-likelihood ratio test.

The mean number of eggs laid by female mosquitoes after each blood meal was analysed by Tukey’s test to investigate the influence of different larval and adult temperatures, and temperature combinations, on the number of eggs produced by adult females at each temperature regimen. Eggs laid by females remained at the same environmental temperature as the mothers, and the mean proportion of eggs hatching at each environmental temperature (23 °C, 27 °C, and 31 °C) were compared using a Mann-Whitney test. The mean number of days between blood meal intake and egg laying, and between oviposition and hatching, were compared using the Mantel-Cox test for pair-wise comparisons between temperatures and by log-rank test for the comparison over all temperatures.

#### Adult female size

Adult wing length was used as a proxy for body size [28–32]. The length of both wings was measured and their arithmetic mean taken as the ‘size’ of each mosquito. A one-way analysis of variance (ANOVA) was used to determine the effect of larval temperature on adult wing length.

All analyses were carried out using R, Version 2.10.1 (R Foundation for Statistical Computing, 2009) [33]. Since the test statistics and their values are provided in the tables of the main text or the Additional file 1, only the corresponding *p*-values are given in the description of the results below.

## Results

### The effect of larval environmental temperature on larval developmental rates and size

**Fig. 1:**
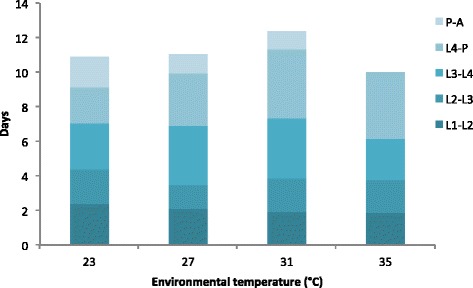
Cumulative number of days to development of each life stage in *Anopheles gambiae* s.s.. Larval stages are L1, L2, L3 and L4; pupae are denoted by P and adults by A. Larvae kept at 35 ± 1 °C died, so that imagoes did not emerge from their pupal cases at this temperature

**Fig. 2:**
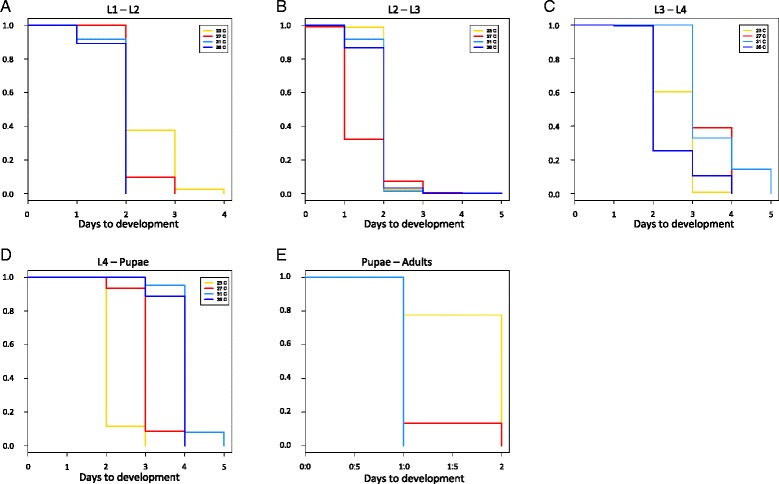
Kaplan-Meier plots of the stage-specific survival analysis of development times. **a** Development time from L1 to L2 larvae. **b** Development time from L2 to L3 larvae. **c** Development time from L3 to L4 larvae. **d** Development time from L4 larvae to pupae. **e** Development time from pupae to adults. Coloured lines represent the environmental temperatures investigated as described in Fig. 1

When males and females are combined, higher temperature from 23 °C to 31 °C slowed the development from hatching to emerging (Fig. 1). No mosquitoes emerged at 35 °C. The effect of temperature differed among the larval stages. Increasing temperature increased the developmental rates of first instar larvae and of pupae, but decreased the developmental rates of fourth instar larvae. Considering only the female mosquitoes, the age at emergence increased with increasing temperature from 10.92 days at 23 °C to 12.35 days at 31 °C. The results of the Kaplan-Meier analyses are presented in Fig. 2.

**Fig. 3:**
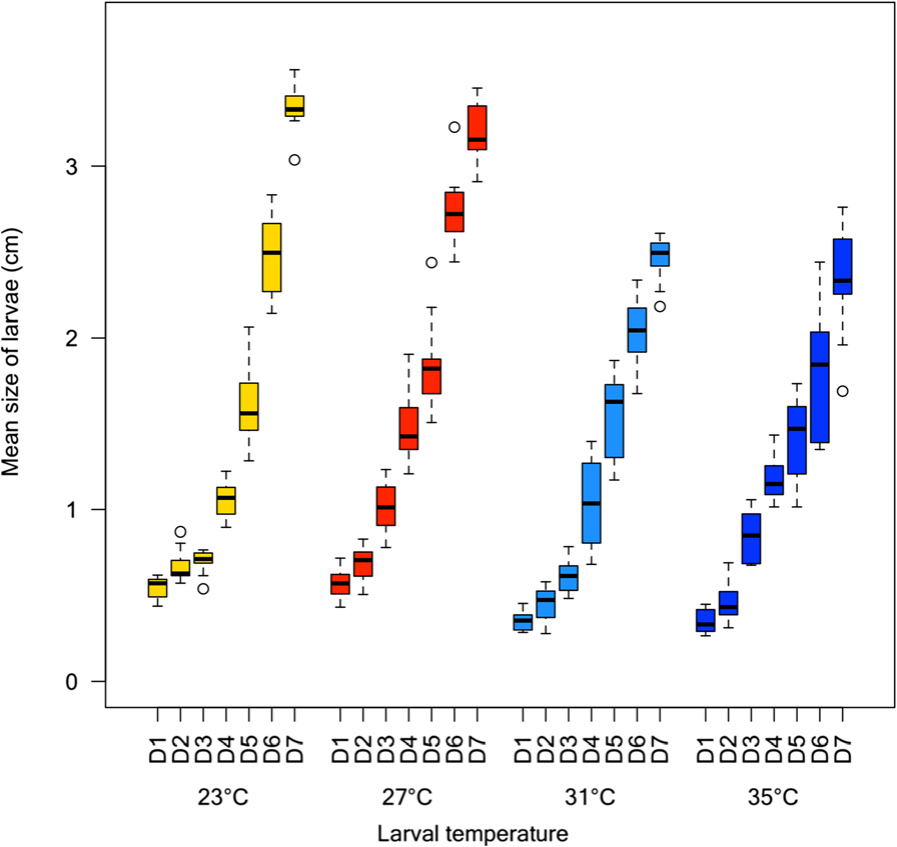
Mean larval size by day as a function of environmental temperature. Size of larvae (in cm) by day post-hatching (D1 is 24 h post-hatching, D2 48 h post-hatching, etc.) and by temperature (23 ± 1 °C: yellow; 27 ± 1 °C: red; 31 ± 1 °C: light blue; 35 ± 1 °C: dark blue). The bottom and top of the boxes describe the first and third quartiles respectively, and the bold line inside the boxes represents the median. The whiskers show the data minima and maxima, and the small circles represent outliers

Figure 3 shows the daily increase in larval size (averaging across all larval stages, from L1 to L4) at all four environmental temperatures for seven days, by which time all larvae pupated. The overall effect of temperature on larval size was statistically significant (*p* = 0.005). Higher temperatures resulted, overall, in smaller larvae on days 1 (*p* < 0.001), 2 (*p* < 0.001), 4 (*p* < 0.001), 5 (*p* = 0.006), 6 (*p* < 0.001) and 7 (*p* < 0.001), with an increase observed on day 3 (*p* < 0.001). The values of the pair-wise comparisons between mean larval sizes for larvae reared at every temperature regimen, as well as test statistics for the overall comparisons across temperatures are given in Additional file 1: Table S1.

### The effect of larval and adult environmental temperature on blood feeding rates

The temperature at which adult mosquitoes were reared as larvae showed no statistically significant effect on the probability of females taking a second blood meal, although females reared at 31 °C were significantly less likely to take a third blood meal than females reared at 23 °C (*p* < 0.001) and females reared at 27 °C (*p* = 0.002) (Additional file 1: Table S2).

All females fed the first time they were offered a blood meal. However, the environmental temperature at which adults were kept had a significant effect on the probability of the mosquitoes feeding for a second and third time (Table 1). For the second blood meal, increasing the temperature from 23 °C to 27 °C decreased the probability of feeding from 0.86 to 0.76 (*p* = 0.049), as did an increase from 23 °C (0.86 probability of blood feeding) to 31 °C (0.64 probability of blood feeding) (*p* < 0.001). An increase from 27 °C to 31 °C did not significantly reduce feeding rates (0.76 at 27 °C vs. 0.64 at 31 °C (*p* = 0.065). For the third blood meal, increases from 23 °C to 27 °C, 27 °C to 31 °C, and 23 °C to 31° all significantly decreased the probability of females feeding on blood (0.48 vs. 0.22, *p* < 0.001; 0.22 vs.0.04, *p* = 0.002; and 0.48 vs.0.04, *p* < 0.001, respectively).

**Table 1:** Two-group comparisons and overall trend of the effect of increasing adult environmental temperature on the odds of blood feeding

	Test statistic	27 °C ± 1 (with respect to 23 °C)	31 °C ± 1 (with respect to 23 °C)	31 °C ± 1 (with respect to 27 °C)
2^nd^ blood meal	Log odds of feeding	−0.706	−1.272	−0.566
	log-likelihood ratio test	−1.972	−3.509	−1.844
	*p*-value	0.049	<0.001	0.065
3^rd^ blood meal	Log odds of feeding	−1.188	−2.959	−1.771
	log-likelihood ratio test	−4.004	−5.397	−3.177
	*p*-value	<0.001	<0.001	0.0015

### The effect of larval and adult environmental temperature on egg laying, number of eggs laid, and egg hatching

Rearing larvae at higher temperatures significantly decreased the probability of egg laying for adult females kept at 23 °C and 27 °C, but not for adults kept at 31 °C, after the first two blood meals (Table 2; all females laid eggs after the third blood meal). The temperature of the adult environment had no effect on the probability of female mosquitoes laying eggs after their first or third blood meal; however, after the second blood meal, an increase from 27 °C to 31 °C and from 23 °C to 31 °C resulted in a significantly lower probability of laying eggs (0.65 vs. 0.46, *p* = 0.022, and 0.72 vs. 0.46, *p* = 0.002, respectively).

**Table 2:** Two-group comparisons and overall trend of the effect of increasing larval environmental temperature on the odds of laying eggs by *Anopheles gambiae* s.s. females kept at all adult temperatures for the first two blood meals^a^

			Larval temperature (°C)		
	Adult temperature (°C)		27 ± 1 (with respect to 23 °C)	31 ± 1 (with respect to 23 °C)	31 ± 1 (with respect to 27 °C)
1^st^ blood meal		Log odds of laying eggs	−0.783^b^	−1.658	−0.876
	23 ± 1	log-likelihood ratio test	−1.187	−2.455	−1.515
		*p*-value	0.235	0.014	0.130
	27 ± 1	Log odds of laying eggs	−2.565	−4.069	−1.504
		log-likelihood ratio test	−2.383	−3.828	−3.086
		*p*-value	0.017	<0.001	0.002
	31 ± 1	Log odds of laying eggs	0.767^c^	−0.028	−0.795
		log-likelihood ratio test	1.386	−0.048	−1.392
		*p*-value	0.166	0.962	0.164
2^nd^ blood meal		Log odds of laying eggs	−1.952	−3.312	−1.36
	23 ± 1	log-likelihood ratio test	−2.398	−3.805	−2.276
		*p*-value	0.016	<0.001	0.023
	27 ± 1	Log odds of laying eggs	−1.161	−2.496	−1.335
		log-likelihood ratio test	−1.613	−3.486	−2.534
		*p*-value	0.107	<0.001	0.011
	31 ± 1	Log odds of laying eggs	1.121	0.359	−0.762
		log-likelihood ratio test	1.557	0.465	−1.218
		*p*-value	0.120	0.642	0.223

^a^All females laid eggs after the third blood meal. ^b^A negative value of the log odds represents a decrease in the proportion of females laying eggs with respect to the reference temperature, whilst a positive value^c^ represents an increase in egg laying rate

The temperature at which mothers were reared as larvae significantly influenced the number of eggs laid at 23 °C and 31 °C after the first blood meal, but had no effect at 27 °C – a similar result to the effect of temperature on the time to egg laying (see below). After the second blood meal, females reared at 27 °C and 31 °C laid significantly fewer eggs than those reared at 23 °C among adults kept at 23 °C. Females reared at 31 °C also laid fewer eggs than females reared at 27 °C among adults kept at 27 °C. Among adult mosquitoes at 23 °C, females reared at 27 °C also laid significantly fewer eggs than females reared at 23 °C. The temperature at which females were reared as larvae had no impact on the number of eggs laid after the third blood meal (Table 3).

**Table 3:** Two-group comparisons and overall trend of the effect of increasing larval environmental temperature on the mean number of eggs laid by adult *Anopheles gambiae* s.s. females (reared from these larvae) kept at all adult temperatures. No mosquitoes held at 31 °C survived long enough to blood feed for a third time

	Adult temperature (°C)		Larval temperature (°C)			
			23 ± 1	27 ± 1 (with respect to 23 °C)	31 ± 1 (with respect to 23 °C)	31 ± 1 (with respect to 27 °C)
1^st^ blood meal		Mean n° of eggs (±SD)	53.11 (2.66)	41.39 (2.98)	22.31 (5.79)	22.31 (5.79)
	23 ± 1	Test statistic (*p*-value)		2.941 (*p* < 0.05)	5.501 (*p* < 0.01)	3.212 (*p* < 0.05)
	27 ± 1	Mean n° of eggs (±SD)	48.67 (2.86)	52.77 (4.09)	48.35 (5.14)	
		Test statistic (*p*-value)		−0.846 (0.4)	0.057 (0.95)	0.662 (0.51)
	31 ± 1	Mean n° of eggs (±SD)	39.00 (3.9)	23.17 (2.77)	14.29 (3.38)	
		Test statistic (*p*-value)		3.337 (*p* < 0.01)	4.719 (*p* < 0.001)	1.899 (0.064)
2^nd^ blood meal		Mean n° of eggs (±SD)	57.53 (3.23)	38.6 (3.55)	30.71 (2.21)	
	23 ± 1	Test statistic (*p*-value)		3.929 (*p* < 0.001)	3.803 (*p* < 0.001)	1.146 (0.261)
	27 ± 1	Mean n° of eggs (±SD)	46.57 (5.57)	57.96 (4.60)	28.17 (8.05)	28.17 (8.05)
		Test statistic (*p*-value)		−1.584 (0.12)	1.907 (0.065)	3.455 (*p* < 0.01)
	31 ± 1	Mean n° of eggs (±SD)	29.75 (4.23)	13.73 (4.19)	22.00 (5.71)	
		Test statistic (*p*-value)		1.88 (0.077)	0.932 (0.376)	−1.136 (0.269)
3^rd^ blood meal		Mean n° of eggs (±SD)	50.54 (2.47)	44.6 (2.58)	36.00 (3.96)	
	23 ± 1	Test statistic (*p*-value)		1.657 (0.1)	2.535 (*p* < 0.05)	1.548 (0.135)
	27 ± 1	Mean n° of eggs (±SD)	54.18 (7.08)	47.20 (6.60)	50.75 (28.21)	
		Test statistic (*p*-value)		0.76 (0.457)	0.173 (0.866)	−0.186 (0.855)

**Fig. 4:**
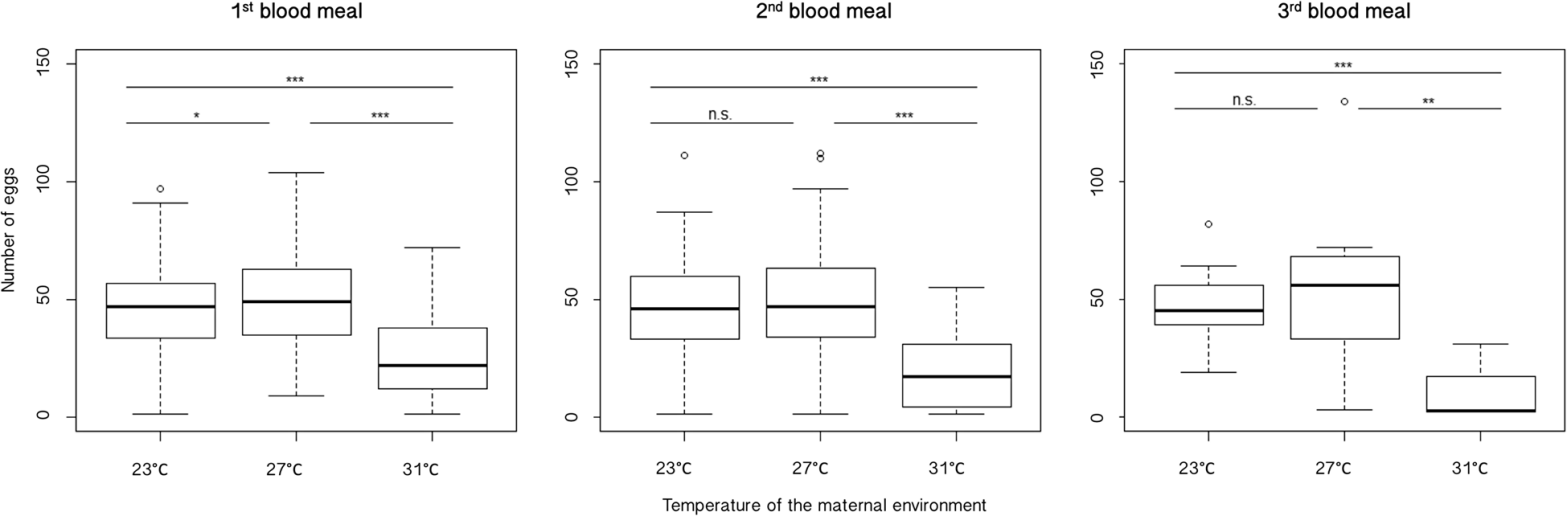
Number of eggs laid at each adult temperature after each blood meal. The bottom and top of the boxes describe the first and third quartiles respectively, and the bold line inside the boxes represents the median. The whiskers show the data minima and maxima, and the small circles represent outliers. (n.s.: not significant; *: *p* < 0.05; **: *p* < 0.01; ***: *p* < 0.001)

Figure 4 shows the effect of the environmental maintenance temperature of adult females on the number of eggs laid after each blood meal. After the first blood meal, females held at 23 °C and 27 °C laid 43 ± 2 eggs (mean ± SE) and 50 ± 2 eggs (*p* = 0.036) respectively, while females at 31 °C laid significantly fewer eggs than females at both of the other regimens (25 ± 2 eggs; *p* < 0.001 in both comparisons). After the second blood meal, females at 23 °C and 27 °C laid 47 ± 2 eggs and 47 ± 4 eggs (*p* = 0.953) respectively, while females at 31 °C laid 18 ± 3 eggs (*p* < 0.001). After the third blood meal, females at 23 °C and 27 °C laid 47 ± 2 eggs and 51 ± 6 eggs (*p* = 0.365), whereas females at 31 °C laid 10 ± 7 eggs (*p* < 0.001 with respect to 23 °C and *p* = 0.007 with respect to 27 °C).

Larval rearing temperatures of mothers had no effect on the mean proportion of laid eggs that hatched into larvae for the first and third blood meals. After the second blood meal, the mean proportion of eggs laid by females reared at 27 °C and 31 °C and that hatched, was significantly higher in comparison with the hatching rate of those eggs laid by females reared at 23 °C (Additional file 1: Table S2). Because the larval temperature at which the mothers had been reared had no impact otherwise on the mean proportion of eggs that hatched, this is likely to be a chance event. The temperature at which the females spent their adult life did impact the proportion of offspring hatching after the second and third blood meal (Table 4).

**Table 4:** Effect of adult environmental temperature on the proportion of laid eggs that hatched after each blood meal taken by *Anopheles gambiae* s.s.

		23 ± 1 °C	27 ± 1 °C (with respect to 23 °C)	31 ± 1 °C (with respect to 23 °C)	31 ± 1 °C (with respect to 27 °C)
1^st^ blood meal	Proportion	0.960	0.964	0.961	
	Test statistic (*p*-value)		−1 (0.757)	−0.17 (0.690)	0.58 (0.825)
2^nd^ blood meal	Proportion	0.890	0.903	0.742	
	Test statistic (*p*-value)		−1.63 (0.784)	8.73 (0.007)	9.78 (0.019)
3^rd^ blood meal	Proportion	0.810	0.795	0.632	
	Test statistic (*p*-value)		1.07 (0.315)	2.76 (0.010)	2.44 (0.043)

### Time to egg laying and time to egg hatching

Table 5 shows the effect of the environmental temperature at which larvae were reared on the time between blood feeding and egg laying of the resulting female mosquitoes, for each blood meal. The temperature at which females were reared as larvae influenced the time to egg laying following the first blood meal only, when an increase in larval environmental temperature from 23 °C to 27 °C and from 23 °C to 31 °C resulted in a significantly longer gonotrophic development (from 4.03 to 4.15 days (*p* = 0.03) and from 4.03 to 4.29 days (*p* < 0.001), respectively). Larval temperature did not influence the duration of time for females to lay eggs after the second (average duration of 4.0 days) or third blood meal (average duration of 3.45 days). At 23 °C, the average duration of time for females to lay eggs was significantly shorter after the third blood meal (3.57 days) than after the first (4.03 days, *p* = 0.001) or second blood meal (4.0 days, *p* = 0.02). At 27 °C, the time to oviposition was also significantly shorter after the third blood meal (3.31 days) than after the first (4.15 days, *p* < 0.001) or second blood meal (4.03 days, *p* < 0.001). At 31 °C, the time to egg laying was significantly shorter after the second blood meal (4.0 days) than after the first blood meal (4.29 days, *p* = 0.004), and after the third blood meal (3.44 days) than after the first blood meal (4.29 days, *p* < 0.001), but there was no significant difference between the third (3.44 days) and second blood meal (4.0 days, *p* = 0.057).

**Table 5:** Two-group comparisons and overall trend of the effect of increasing larval environmental temperature on the time to egg laying by adult *Anopheles gambiae* s.s. females reared from the larvae

		23 °C ± 1	27 °C ± 1 (with respect to 23 °C)	31 °C ± 1 (with respect to 23 °C)	31 °C ± 1 (with respect to 27 °C)	Overall effect of larval temperature on time to egg laying	
1^st^ blood meal	Days (±SD)	4.03 (±0.18)	4.15 (±0.36)	4.29 (±0.46)			
	Mantel-Cox test statistic		4.51	11.08	2.20	Log-rank test statistic	11.06
	*p*-value		0.03	<0.001	0.14	*p*-value	<0.001
2^nd^ blood meal	Days (±SD)	4.00	4.03 (±0.18)	4.00 (±0.0)			
	Mantel-Cox test statistic		0.48	0	0.50	Log-rank test statistic	0.05
	*p*-value		0.49	0.99	0.48	*p*-value	0.83
3^rd^ blood meal	Days (±SD)	3.57 (±0.5)	3.31 (±0.53)	3.44 (±0.53)			
	Mantel-Cox test statistic		2.02	0.22	0.19	Log-rank test statistic	2.03
	*p*-value		0.16	0.64	0.67	*p*-value	0.36

Rearing adults at warmer temperatures had no effect on the time to oviposition for the first, second, or third batch of eggs (Additional file 1: Table S3). However, at all adult environmental temperatures considered, the duration between blood feeding and oviposition became significantly shorter after each blood meal (23 °C: *p* < 0.001; 27 °C: *p* < 0.001; 31 °C: *p* = 0.027; Additional file 1: Table S4).

The effect of the environmental temperatures on time to eggs hatching is shown in Table 6. Keeping the eggs at warmer temperatures significantly quickened to hatching of the first and second batch of eggs (*p* < 0.001 in both cases), but a temperature of 31 °C had no effect on the time to hatching of the eggs produced by the third blood meal (*p* = 0.69).

**Table 6:** Two-group comparisons and overall trend of the effect of increasing the environmental temperature of eggs on their time to hatching

		23 ± 1 °C	27 ± 1 °C (with respect to 23 °C)	31 ± 1 °C (with respect to 23 °C)	31 ± 1 °C (with respect to 27 °C)	Overall effect of larval temperature on time to egg hatching	
1^st^ blood meal	Days (±SD)	1.72 (±0.48)	1.07 (±0.26)	1.75 (±0.81)			
	Mantel-Cox test statistic		61.88	0.05	41.05	Log-rank test statistic	78.49
	*p*-value		<0.001	0.82	<0.001	*p*-value	<0.001
2^nd^ blood meal	Days (±SD)	2.43 (±0.65)	1.83 (±0.62)	1.92 (±0.49)			
	Mantel-Cox test statistic		17.71	12.81	0.01	Log-rank test statistic	21.37
	*p*-value		<0.001	<0.001	0.91	*p*-value	<0.001
3^rd^ blood meal	Days (±SD)	2.89 (±0.83)	2.45 (±1.1)	3 (±0.0)			
	Mantel-Cox test statistic		0.75	0.03	0.02	Log-rank test statistic	0.75
	*p*-value		0.39	0.87	0.9	*p*-value	0.69

For eggs produced after the first blood meal, a 4 °C increase in the environmental temperature of the eggs from 23 °C to 27 °C significantly shortened the time to hatching from an average of 1.72 days to 1.07 days (*p* < 0.001), but a 4 °C increase from 27 °C to 31 °C increased the time to hatching from 1.07 to 1.75 days (*p* < 0.001). The difference in hatching time between the coldest (23 °C) and the warmest environmental temperature (31 °C) was not significant (*p* = 0.82).

After the second blood meal, a 4 °C increase from 23 °C to 27 °C significantly shortened the time to hatching, from 2.15 days to 2.11 days (*p* < 0.001), whereas a 4 °C increase from 27 °C to 31 °C had no effect on the time to hatching (2.11 days and 2 days respectively, *p* = 0.91). The 8 °C increase from 23 °C to 31 °C, therefore, also significantly shortened the time to hatching (*p* < 0.001).

The temperature at which adult females were reared as larvae was found to have no effect on the time to hatching of their eggs (Additional file 1: Table S5).

### The effect of larval environmental temperature on adult mosquito size

**Fig. 5:**
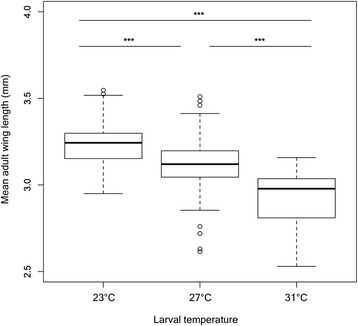
Adult wing length (mm) as a function of larval environmental temperature. The bottom and top of the boxes describe the first and third quartiles respectively, and the bold line inside the boxes represents the median. The whiskers show the data minima and maxima, and the small circles represent outliers. (***: *p* < 0.001)

The mean wing lengths of adult mosquitoes reared at 23 °C, 27 °C, or 31 °C as larvae are shown in Fig. 5. There is a significant trend for the size of adult female mosquitoes to decrease with increasing larval environmental temperature (overall *p* < 0.001). Every increase in larval temperature brought about a significant decrease in size (27 °C vs. 23 °C; 31 °C vs. 23 °C; and 31 °C vs. 27 °C, all *p*-values <0.001; Additional file 1: Table S6). Temperature of the adult environment was found not to have any effect on the wing length of adult mosquitoes (F-statistic = 0.17, *p* = 0.844).

## Discussion

The temperature experienced by *Anopheles gambiae* s.s. during all of its life stages, from eggs through to adults, is shown here to affect several key life-history parameters of considerable importance for a better understanding of the impact of climatic variables on mosquito population dynamics, namely, larval and adult size, development rate of each life stage, propensity to blood feed, number of eggs laid (fecundity) and proportion of these that hatch (fertility). Our results complement, through more detailed experiments, scarce studies in this area and, importantly, examine the question of how the environmental temperature of the immature, aquatic stages, may influence life-history traits of the adult, aerial stages.

Our results confirm those of previous studies [13, 34, 35] reporting that the temperature of the larval environment significantly influences both larval and adult size, with higher temperatures resulting in smaller larvae and adults. This has important implications for predictions of malaria spread under warmer climate scenarios, as the size of *Anopheles gambiae* s.s. has been shown to be an important determinant of its vectorial capacity for malaria transmission [36]. However, to our knowledge, there are currently very few data on the influence of environmental temperature during both the juvenile and adult stages on the life-history parameters of *Anopheles gambiae* s.s..

The average time spent in each immature stage was influenced by environmental temperature, but the direction of the effect was different for the earlier (shortening the time until the L3–L4 stage transition) and later stages (lengthening the time from L3–L4 larvae to imagoes), with the net result that higher temperatures resulted in a longer development time from egg to imago. Bayoh and Lindsay [23] indicated that increasing temperature results in faster development rates which peak, in their case, at 28 °C. Under our experimental conditions, we observed the fastest development rate at 31 °C. Bayoh and Lindsay [23] also remarked that no imagoes emerged at temperatures above 34 °C, which agrees with our findings that larvae and pupae reared at 35 °C died before they could develop into adults.

Our data also suggest there may be an optimal temperature for the gonotrophic development of *Anopheles gambiae* s.s. egg batches. Adults which emerged from immatures kept at 27 °C, and which were themselves kept at 27 °C, laid the most eggs, whereas those reared and kept at higher (and lower) temperatures laid significantly fewer eggs. Interestingly, the temperature at which mothers had been reared as larvae influenced the numbers of eggs laid after the first two blood meals, suggesting that this was not simply an effect of adult size. Higher adult temperatures resulted in a lower probability of adult females taking blood meals. When temperature affected fecundity, higher temperatures also decreased the probability of laying eggs.

Overall, increasing adult temperature did not influence the duration of the time between blood feeding and egg laying, but the duration of the gonotrophic development was significantly shortened with each additional blood meal for each temperature. The environmental temperature at which the eggs were kept did not influence the time to hatching in a discernible trend, in keeping with [37] and suggesting that while temperature may affect the time to egg hatching, the effect is minimal within an optimal range for egg development (24-30 °C), which includes the temperatures considered here.

Previous studies have examined the relationship between temperature and the development rate of other *Anopheles* species [11, 13, 22, 35, 38], as well as other mosquito vectors [39]. Research elsewhere has also looked at the effect of temperature on the gonotrophic cycle, fecundity, hatching rate, and sex ratio [35, 40], as well as the relationship between larval food quantity and several life-history parameters [41]. Our results agree with those of [37], showing that temperature can influence both the time to hatching and hatching counts.

The experimental design presented here did not account for the diurnal and nocturnal fluctuations in temperature and humidity that would affect mosquito development and survival in the field. The effects of other climatic and environmental factors on *An. gambiae* reproductive and life-history parameters, as well as the influence of local air temperature fluctuations on the water temperature of mosquito breeding sites [42, 43], require further research. In addition, *Anopheles gambiae* s.s. is only one of the seven major vectors of human malaria in Africa [44] and available data on the sensitivity of the other species to climatic variables such as temperature, as well as population-related factors, are even more scarce than data on *Anopheles gambiae* s.s.. Climate change and global warming are expected to influence the reproductive and life-history parameters [22] of different mosquito species in different ways [45]. More comprehensive data specific to each malaria vector species are required to define the dependency of mosquitoes’ population dynamics on climate-related variables, which, combined with regional climate data predictions, will allow sound and dependable forecasting of disease vector population dynamics and transmission patterns.

### Conclusions

There is currently considerable uncertainty about how climate change will affect temperatures and temperature fluctuations, and how this will, in turn, impact the population dynamics of disease vectors. An understanding of how short- and long-term climate change-induced temperature variations will affect the life-history parameters of disease vectors and influence their population dynamics and geographical spread is necessary for more robust and dependable forecasts of disease transmission. To this end, the results of the experimental work described here have been recently used to parameterise and fit mathematical models of *Anopheles gambiae* s.s. population dynamics to mosquito abundance data. This is a first step towards the development of more detailed and robust frameworks to better understand the effects of a warmer world on the distribution and density of mosquito vectors of disease.

## Additional file

Additional file 1:
**Description of mosquito larvae and adult wing size measurements and additional data tables.** (DOC 329 kb)

## Abbreviations

ANOVA: Analysis of variance
RH: Relative humidity
