# Supplementary material for: Larval and adult environmental temperatures influence the adult reproductive traits of Anopheles gambiae s.s
Source: Parasit Vectors. 2015 Sep 17;8:456. doi: 10.1186/s13071-015-1053-5 (PMC4573685; doi:10.1186/s13071-015-1053-5)
Supplement: Additional file 1: — Description of mosquito larvae and adult wing size measurements and additional data tables. (DOC 329 kb) [file 13071_2015_1053_MOESM1_ESM.doc]

**Supplementary Files**

**Larval and adult environmental temperatures influence the adult reproductive traits of *Anopheles gambiae* s.s.**

Céline D. Christiansen-Jucht^1*^, Paul E. Parham^2^, Adam Saddler^3^, Jacob C. Koella^4^, and María-Gloria Basáñez^1^

1 Department of Infectious Disease Epidemiology, School of Public Health, Faculty of Medicine (St Mary’s campus), Imperial College London, Norfolk Place, London W2 1PG, UK

2 Department of Public Health and Policy, Faculty of Health and Life Sciences, University of Liverpool, 33 Finsbury Square, London EC2A 1AG, UK

3 Department of Epidemiology and Public Health, Health Interventions Unit, Swiss TPH, Basel, Switzerland

4 Faculté des Sciences, Institut de Biologie, Université de Neuchâtel, Rue Emile-Argand 11, CH-2000 Neuchâtel, Switzerland

**Figure S1. *Anopheles* larvae measurements.**

**
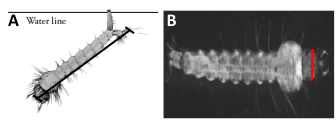
**

**A**, Larval body is measured from the distal tip of the head to the end of the anal segment, excluding all antennae, feeding brush, and caudal hair. **B**, Larval head width (for instar determination) is measured across the head, again excluding caudal hair from the measurement, as shown in red [1].

**Figure S2. Adult female *Anopheles* wing measurement.**

**
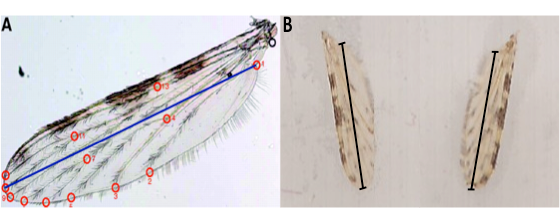
**

Mosquito wing length is measured from the inside of the wing immediately at the base of the node by which it is attached to the mosquito's body to the outer tip of the wing. **A**, Photograph of a wing of *Anopheles gambiae* s.l., with the straight-line wing-length measurement displayed in blue, from [2]. Note the length of the node is not included in wing-length measurement. **B**, Photograph of the left and right wings of an *Anopheles gambiae* s.s. mosquito obtained during the work conducted at Imperial College London, Silwood Park campus, with the wing length measurement displayed as the solid black lines.

**Table S1. Two-group comparisons and overall trend of the effect of increasing larval environment temperature on mean larval size across all larval instars (L1 to L4) for seven days prior to pupation in *Anopheles gambiae* s.s.**

|  |  | **23±1°C** | **27±1°C (with respect to 23°C)** | **31±1°C (with respect to 23°C)** | **31±1°C (with respect to 27°C)** | **35±1°C (with respect to 23°C)** | **35±1°C (with respect to 27°C)** | **35±1°C (with respect to 31°C)** | **Overall effect on larval body length** | | | |
| --- | --- | --- | --- | --- | --- | --- | --- | --- | --- | --- | --- | --- |
| **Day 1** | Size (± SE) (mm) | 5.47 (0.21) | 5.64 (0.29) | 3.53 (0.18) | | 3.50 (0.21) | | |  | | | |
|  | Tukey test statistic |  | 0.24 | 49.12 | 38.20 | 43.90 | 35.86 | 0.03 | F statistic | 27.20 | | |
|  | p-value |  | 0.63 | <0.001 | <0.001 | <0.001 | <0.001 | 0.86 | p-value | <0.001 | | |
| **Day 2** | Size (± SE) (mm) | 6.75 (0.30) | 6.91 (0.30) | 4.54 (0.31) | | 4.62 (0.39) | | |  | | | |
|  | Tukey test statistic |  | 0.15 | 25.93 | 30.03 | 18.88 | 21.95 | 0.03 | F statistic | 15.85 | | |
|  | p-value |  | 0.71 | <0.001 | <0.001 | <0.001 | 0.001 | 0.87 | p-value | <0.001 | | |
| **Day 3** | Size (± SE) (mm) | 6.99 (0.22) | 10.13 (0.47) | 6.16 (0.32) | | 8.45 (0.50) | | |  | | | |
|  | Tukey test statistic |  | 36.73 | 4.64 | 49.53 | 7.15 | 6.11 | 15.12 | F statistic | | 19.81 | |
|  | p-value |  | <0.001 | 0.04 | <0.001 | 0.02 | 0.02 | 0.001 | p-value | | <0.001 | |
| **Day 4** | Size (± SE) (mm) | 10.54 (0.33) | 14.71 (0.68) | 10.35 (0.76) | | 11.87 (0.43) | | |  | | | |
|  | Tukey test statistic |  | 30.26 | 0.05 | 18.07 | 6.12 | 12.32 | 3.02 | F statistic | | 12.06 | |
|  | p-value |  | <0.001 | 0.83 | <0.001 | 0.02 | 0.003 | 0.10 | p-value | | <0.001 | |
| **Day 5** | Size (± SE) (mm) | 16.24 (0.83) | 18.46 (0.88) | 15.50 (0.81) | | 14.15 (0.73) | | |  | | | |
|  | Tukey test statistic |  | 3.38 | 0.41 | 6.16 | 3.56 | 14.2 | 1.51 | F statistic | | | 4.9 |
|  | p-value |  | 0.08 | 0.53 | 0.02 | 0.08 | 0.001 | 0.23 | p-value | | | 0.006 |

**Table S1. Continued. Two-group comparisons and overall trend of the effect of increasing larval environment temperature on mean larval size across all larval instars (L1 to L4) for seven days prior to pupation in *Anopheles gambiae* s.s.**

| **Day 6** | Size (± SE) (mm) | 24.83 (0.73) | 27.41 (0.70) | 20.28 (0.71) | | | 17.85 (1.20) | | | |  | |
| --- | --- | --- | --- | --- | --- | --- | --- | --- | --- | --- | --- | --- |
|  | Tukey test |  | 6.58 | 20.02 | | 51.19 | 24.82 | 47.56 | | 3.03 | F statistic | 25.28 |
|  | p-value |  | 0.02 | <0.001 | | <0.001 | <0.001 | <0.001 | | 0.10 | p-value | <0.001 |
| **Day 7** | Size (± SE) (mm) | 33.44 (0.45) | 31.86 (0.57) | 24.55 (0.43) | | | 23.26 (0.99) | | | |  | |
|  | Tukey test |  | 4.63 | 200.10 | 103.20 | | 86.45 | | 56.00 | 1.41 | F statistic | 61.03 |
|  | p-value |  | 0.05 | <0.001 | <0.001 | | <0.001 | | <0.001 | 0.25 | p-value | <0.001 |
| **Overall** | | | | | | | | | | | F statistic | 4.43 |
|  |  |  |  |  |  |  |  |  |  |  | p-value | 0.005 |

**Table S2. Comparison by logistic regression of the effect of larval temperature on the propensity to feed of female *Anopheles gambiae* s.s..**

|  | **Test statistic** | **27°C±1 (with respect to 23°C)** | **31°C±1 (with respect to 23°C)** | **31°C±1 (with respect to 27°C)** |
| --- | --- | --- | --- | --- |
| **2^nd^ blood meal** | Log odds of feeding | 0.562 | 0.45 | -0.111 |
|  | log-likelihood ratio test | 1.803 | 1.344 | -0.322 |
|  | p-value | 0.071 | 0.179 | 0.748 |
| **3^rd^ blood meal** | Log odds of feeding | -0.194 | -1.466 | -1.272 |
|  | log-likelihood ratio test | -0.673 | -3.591 | -3.139 |
|  | p-value | 0.501 | <0.001 | 0.002 |

**Table S3. Effect of larval temperature on the proportion of laid eggs that hatched after each blood meal taken by *Anopheles gambiae* s.s. adult females reared from these larvae and kept at each of the adult temperatures.** No mosquitoes survived at 31°C long enough to blood feed for a third time.

|  |  |  | **Larval temperature (°C)** | | | |
| --- | --- | --- | --- | --- | --- | --- |
|  | **Adult temperature (°C)** |  | **23±1°C** | **27±1 (with respect to 23°C)** | **31±1 (with respect to 23°C)** | **31±1 (with respect to 27°C)** |
| **1^st^ blood meal** | **23±1** | Proportion | 0.964 | 0.973 | 0.879 | |
|  |  | Test statistic (p-value) |  | -1.36 (0.292) | 6.31 (0.362) | 6.97 (0.831) |
|  | **27±1** | Proportion | 0.958 | 0.969 | 0.971 | |
|  |  | Test statistic (p-value) |  | -1.76 (0.402) | -1.64 (0.806) | -9.94 (0.528) |
|  | **31±1** | Proportion | 0.972 | 0.963 | 0.920 | |
|  |  | Test statistic (p-value) |  | 0.97 (0.766) | 3.21 (0.336) | 2.43 (0.464) |
| **2^nd^ blood meal** | **23±1** | Proportion | 0.940 | 0.850 | 0.637 | |
|  |  | Test statistic (p-value) |  | 7.92 (0.015) | 14.59 (0.007) | 7.2 (0.242) |
|  | **27±1** | Proportion | 0.882 | 0.925 | 0.879 | |
|  |  | Test statistic (p-value) |  | -3.56 (0.763) | 0.17 (0.130) | 2.71 (0.078) |
|  | **31±1** | Proportion | 0.485 | 0.779 | 0.864 | |
|  |  | Test statistic (p-value) |  | -5.15 (0.481) | -6.54 (0.217) | -2.04 (0.62) |
| **3^rd^ blood meal** | **23±1** | Proportion | 0.842 | 0.773 | 0.778 | |
|  |  | Test statistic (p-value) |  | 4 (0.145) | 2.15 (0.352) | -0.15 (0.865) |
|  | **27±1** | Proportion | 0.756 | 0.811 | 0.872 | |
|  |  | Test statistic (p-value) |  | -2.19 (0.375) | -3.49 (0.792) | -1.27 (0.566) |

**Table S4. Two-group comparisons (Mantel-Cox test) and overall trend (log-rank test) of the effect of increasing adult environmental temperature on the time to laying eggs by adult *Anopheles gambiae*** s.s. females.

| **Blood meal** |  | **23°C±1** | **27°C±1 (with respect to 23°C)** | **31°C±1 (with respect to 23°C)** | **31°C±1 (with respect to 27°C)** | **Overall effect of adult temperature on time to egg laying** | |
| --- | --- | --- | --- | --- | --- | --- | --- |
| **1^st^ Blood meal** | Days (±SD) | 4.11 (±0.31) | 4.12 (±0.32) | 4.18 (±0.39) | 4.18 (±0.39) |  | |
|  | Mantel-Cox test statistic  (p-value) |  | 0.02  (0.90) | 0.88  (0.35) | 0.69  (0.41) | Log-rank test statistic  (p-value) | 0.84  (0.36) |
| **2^nd^ Blood meal** | Days (±SD) | 4.00 (±0.0) | 4.00 (±0.0) | 4.08 (±0.27) | 4.08 (±0.27) |  | |
|  | Mantel-Cox test statistic  (p-value) |  | 0.00  (0.99) | 1.43  (0.23) | 1.37  (0.24) | Log-rank test statistic  (p-value) | 1.29  (0.26) |
| **3^rd^ Blood meal** | Days (±SD) | 3.47 (±0.50) | 3.46 (±0.51) | 3.00 (±0.82) | 3.00 (±0.82) |  | |
|  | Mantel-Cox test statistic  (p-value) |  | 0.00  (0.96) | 0.90  (0.34) | 0.76  (0.38) | Log-rank test statistic  (p-value) | 0.93  (0.63) |

**Table S5. Two-group comparisons (Mantel-Cox test) and overall trend (log-rank test) of the effect of adult environmental temperature on the time between blood feeding and egg laying for three blood meals by *Anopheles gambiae*** s.s.

| **Adult temperature (°C)** |  | **1^st^ blood meal** | **2^nd^ blood meal (with respect to 1^st^)** | **3^rd^ blood meal (with respect to 1^st^)** | **3^rd^ blood meal (with respect to 2^nd^)** | **Overall effect of adult temperature on time to egg laying** | | |
| --- | --- | --- | --- | --- | --- | --- | --- | --- |
| **23±1** | Days (± SD) | 4.11 (±0.315) | 4 (±0.0) | 3.469 (±0.504) | |  | | |
|  | Mantel-Cox test statistic (p-value) |  | 4.18  (0.041) | 27.30  (<0.001) | 12.13  (<0.001) | Log-rank test statistic (p-value) | 27.89  <0.001 | |
| **27±1** | Days (± SD) | 4.118 (±0.324) | 4 (±0.0) | 3.462 (±0.508) | |  | | |
|  | Mantel-Cox test statistic (p-value) |  | 4.45  (0.035) | 20.84  (<0.001) | 8.87  (0.003) | Log-rank test statistic (p-value) | 19.96  <0.001 | |
| **31±1** | Days (± SD) | 4.18 (±0.388) | 4.077 (±0.272) | 3.0 (±0.816) | |  | | |
|  | Mantel-Cox test statistic (p-value) |  | 1.06  (0.30) | 13.69  (<0.001) | 7.87  (0.005) | Log-rank test statistic (p-value) | 4.88  (0.027) | |
| **All adult temperatures** | | | | | Log-rank test statistic  (p-value) | | | 60.02  (<0.001) |

**Table S6. Two-group comparisons (Mantel-Cox test) and overall trend (log-rank test) of the effect of increasing adult environmental temperature on the time to egg hatching for three blood meals by *Anopheles gambiae* s.s.**

|  |  | **23±1°C** | **27±1°C (with respect to 23°C)** | **31±1°C (with respect to 23°C)** | **31±1°C (with respect to 27°C)** | **Overall effect of adult temperature on time to hatching** | |
| --- | --- | --- | --- | --- | --- | --- | --- |
| **1^st^ Blood meal** | Days (±SD) | 1.720 (±0.48) | 1.071 (±0.26) | 1.754 (±0.81) | 1.754 (±0.81) |  | |
|  | Mantel-Cox test (p-value) |  | 61.88  (<0.001) | 0.05  (0.82) | 41.05  (<0.001) | Log rank test (p-value) | 78.49  (<0.001) |
| **2^nd^ Blood meal** | Days (±SD) | 2.433 (±0.65) | 1.827 (±0.62) | 1.92 (±0.49) | 1.92 (±0.49) |  | |
|  | Mantel-Cox test (p-value) |  | 17.71  (<0.001) | 12.82  (<0.001) | 0.01  (0.91) | Log rank test (p-value) | 21.37  (<0.001) |
| **3^rd^ Blood meal** | Days (±SD) | 2.889 (±0.83) | 2.45 (±1.10) | 3.00 (±0.0) | 3.00 (±0.0) |  | |
|  | Mantel-Cox test (p-value) |  | 0.75  (0.39) | 0.03  (0.87) | 0.02  (0.90) | Log rank test (p-value) | 0.75  (0.69) |

**Table S7. Two-group comparisons (Tukey test) and overall trend (F-statistic) of the effect of increasing larval environment temperature on the length of adult female wings in *Anopheles gambiae* s.s.**

|  | **23±1°C** | **27±1°C (with respect to 23°C)** | **31±1°C (with respect to 23°C)** | **31±1°C (with respect to 27°C)** | **Overall effect of larval temperature on adult wing length** | |
| --- | --- | --- | --- | --- | --- | --- |
| Size in mm (± SE) | 3.24 (±0.01) | 3.12 (±0.02) | 2.93 (±0.03) | |  | |
| Tukey test statistic (p-value) |  | 26.93  (<0.001) | 154.57  (<0.001) | 38.51  (<0.001) | F statistic  (p-value) | 65.51  (<0.001) |

**References**

[1] Benedict MQ (2014) Methods in *Anopheles* research. Fourth edition. Atlanta: Centers for Disease Control (CDC), USA. Available: [http://www.mr4.org/Portals/3/MR4_Publications/Methods%20in%20Anopheles%20Research%202014/2014MethodsinAnophelesResearchManualFullVersionv2tso.pdf](http://www.mr4.org/Portals/3/MR4_Publications/Methods in Anopheles Research 2014/2014MethodsinAnophelesResearchManualFullVersionv2tso.pdf) (accessed 20 May 2015)

[2] Huestis DL, Yaro AS, Traoré AI, Adamou A, Kassogué Y, Diallo M (2011) Variation in metabolic rate of *Anopheles gambiae* and *A. arabiensis* in a Sahelian village. J Exp Biol, 214(14): 2345–2353.
